# Measuring early childhood development with The Early Human Capability Index (eHCI): a reliability and validity study in China

**DOI:** 10.1186/s12887-020-02210-w

**Published:** 2020-06-30

**Authors:** Jin Zhao, Sally Anne Brinkman, Yunting Zhang, Yingquan Song, Chunling Lu, Mary Eming Young, Yue Zhang, Patrick Ip, Wenjie Shan, Fan Jiang

**Affiliations:** 1 Child Health Advocacy Institute, Shanghai Children’s Medical Center, Shanghai Jiao Tong University School of Medicine, Shanghai, China; 2 Telethon Kids Institute, University of Western Australia, Perth, Australia; 3 School of Public Health, Faculty of Health and Medical Sciences, University of Adelaide, Adelaide, Australia; 4 China Institute for Educational Finance Research, Peking University, Beijing, China; 5 Division of Global Health Equity, Brigham & Women’s Hospital and Department of Global Health and Social Medicine, Harvard Medical School, Boston, MA 02115 USA; 6 China Development Research Foundation, Center of Child Development, Beijing, China; 7 Children Health Care Department, National Center for Women and Children Health, Chinese Center for Disease Control and Prevention, Beijing, China; 8 Department of Paediatrics and Adolescent Medicine, The University of Hong Kong, Hong Kong, China; 9 Department of Developmental and Behavioral Pediatrics, Shanghai Children’s Medical Center, Shanghai Jiao Tong University School of Medicine, Shanghai, China

**Keywords:** Early childhood development, Measurement, Validation, 3–6

## Abstract

**Background:**

With the importance of early childhood development more recognized by the international society, low-cost and cross-culturally comparable measures of early childhood development is in great demand, both in China and worldwide. In this study, we aim to test the psychometrics of the Chinese version of The Early Human Capability Index (eHCI), which is designed as a measurement for school readiness in large population.

**Methods:**

We evaluated the internal consistency, test-retest reliability, inter-rater reliability, factor structure, criterion-related validity, and discriminant validity of the eHCI in 20,324 preschool children in Shanghai. We also compared eHCI scores with test result of ASQ in 815 children in Yexian and EAP-ECDS in 6947 children in Daming.

**Results:**

The ICC between parents and teachers were 0.83 and 0.63 for Literacy Numeracy and Overall Development. The confirmatory factor analyses showed good model fit (χ2 = 509,323, *p* < 0.001; CFI = 0.901; RMSEA = 0.038). The correlations between the scores of eHCI and other ECD metrics ranged between r = − 0.42 and r = 0.53. The scale discriminated between children’s developmental level based on sex, parental education, family income, family assets, and nutrition status.

**Conclusions:**

Results from Chinese population suggested that eHCI is valid and reliable for measuring early childhood development in children aged 3–6 years. The eHCI can be applied to map the global distribution of early childhood development for allocating scarce resources to help those in greatest demand. Longitudinal studies are warranted to test its predictive validity for later outcomes.

## Background

The importance of early childhood development (ECD) remains profound. The capacities established during early childhood lay the foundation for physical, emotional, and intellectual wellbeing in middle childhood, throughout adolescence and into adulthood, even with multi-generational effects [1]. The 2007 and 2011 Lancet Series on Child Development in Developing Countries spearheaded the review of evidence linking early childhood development with adult health and wellbeing. The 2016 series considered new scientific evidence for intervention, and proposed pathways for implementation of early childhood development at scale [2]. Studies from across the globe, such as the Jamaica project, Perry Preschool and Abecedarian program, have demonstrated that interventions significantly improved childhood development and even later adult outcomes in the studied settings [3–5]. A meta-analysis, however, could not detect large effect sizes for the more recent and larger scale interventions [6], and the study suggests that ability of these measures for detecting effects could be one of the possible explanations. Tools for assessing early development used in small group trials, such as the Griffith and Bayley Scales of Infant Development, may not be effective in evaluating the impact of interventions implemented in large populations [7]. Traditionally, most measures of child development originate from the disciplines of pediatrics or developmental psychology, with focus on screening for developmental disability, which usually accounts for 10–15% of the whole population [8, 9]. However, there is evidence that more than 25% of children experience difficulties in learning, while they were not diagnosed as high-risk population by traditional clinical tools [10]. Moreover, many interventions implemented at scale are aimed at enhancing development, rather than identifying disabilities [11]. Therefore, a high-quality tool for measuring early childhood development is necessary to support the evaluation of early interventions. Such a tool would help to: evaluate children’s comprehensive traits, explore the protective factors that promote development and enhance child development at the population level [12].

Considering the limitations of clinical screening assessments, several tools have emerged to assess early childhood development at the population level. The Caregiver-Reported Early Development Instruments (CREDI) is developed for children under 3 years old and evaluates their early development [13]. As it was designed to function across a wide variety of culture, linguistic, and socioeconomic contexts, it has been promoted in 16 countries. The Early Childhood Development Index (ECDI) was launched by UNICEF as part of the Multiple Indicator Cluster Surveys [14]. It contains 10 items covering the literacy–numeracy, learning, social–emotional, and physical development of children aged three and 4 years. The ECDI has been administrated in more than 60 low- and middle-income countries, and map the global early childhood development status.

Except for those tools developed for children in very early years, the concept of school readiness assessment is also considered to be an important indicator of early childhood development due to its effectiveness as a predictor of children’s future achievement [15]. If children are school ready, then they should be entering the education system with all the skills, capabilities, health and development to take advantage of the school learning environment and improve equity in achieving lifelong learning and full developmental potential among children [16]. The Early Development Instrument (EDI) is one of the few existing measurement that holistically evaluates the school readiness of children aged 3.5–6.5 years [17]. It was well-known as the main assessment tool in the Australian Early Development Census, which is implemented as a developmental census across the entire country once every 3 years [18]. However, the EDI is far from applied in international use, as it was originally designed for western culture. Cultural specificity is a key point in ECD concepts. Different aspects of culture (parenting practices, foods and social norms for example) can be both positive and negative for child development, however western developed instruments do not capture important aspects of child development in the Chinese culture and context. It is essential for any future population monitoring system of child development in China to be based on an instrument adapted to local culture and context. For example, the item of EDI “coming to school dressed appropriately” is intended to assessing children’s ability of organization, but most parents in poor countries and regions have no conditions to purchase “decent clothes” [16]. In view of these limitations, researchers are currently developing new scales that can better reflect child development across different cultures and contexts.

In 2013 the Early Human Capability Index (eHCI) was developed by Brinkman firstly in Tonga for impact evaluation of the school readiness component of the PERAL program [19]. The scale was designed to assess the comprehensive development of children aged 3–6 years at a population-level across diverse cultures. The original Tonga Early Human Capability Index contained 66 items in 9 domains including physical health, general verbal communication, cultural identity and spirituality, social and emotional well-being and skills, perseverance, approaches to learning, numeracy and concepts, formal literacy – reading and formal literacy – writing. It can be filled out by parents, teachers, social workers and other people familiar with the child. eHCI has been applied in other countries in the Pacific, South East Asia and Latin America. In 2014, the process of adapting the eHCI in China commenced. Through a process of discussions with experts in the fields of pediatric medicine and education, the instrument was adapted and revised item by item to conform to the cultural characteristics of China. Following this a series of pilots were conducted with particular attention paid to preventing any ceiling or floor effects for the Chinese population of children aged 3 through to 5 years of age. The aim of this paper is to validate the psychometrics of the Chinese version of the eHCI.

## Methods

### The development of EHCI in Chinese version

**Fig. 1:**
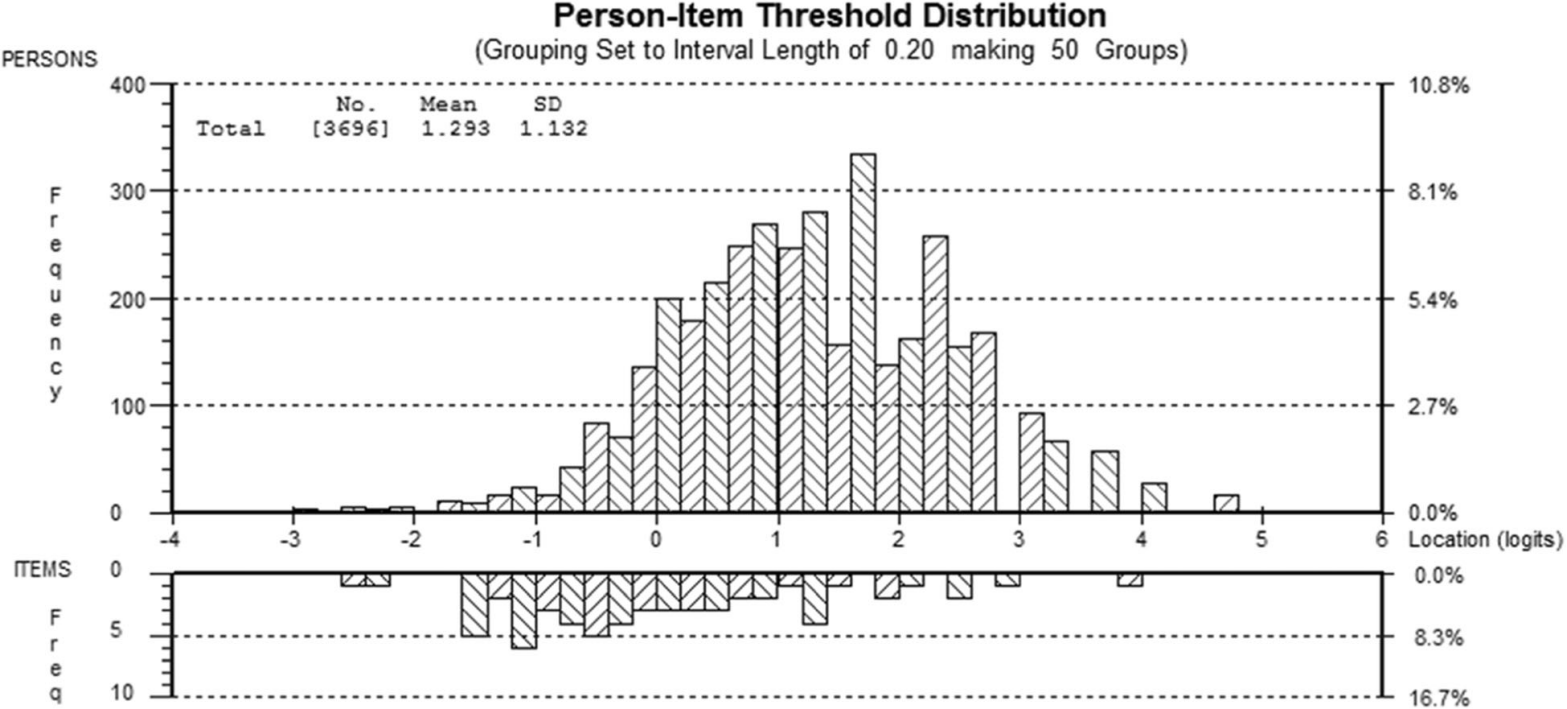
Distribution of item and person locations: all 60 items

In 2014, under the guidance of Brinkman, an early childhood development specialist in Australia, the China Development Research Foundation (CDRF), in collaboration with the Shanghai Children’s Medical Center affiliated to Shanghai Jiaotong University School of Medicine, started working on the Chinese version of the early Human Capacity Index. Through discussions among experts in different fields such as pediatric medicine and education, the team translated each item into Chinese and made necessary amendments to reflect China’s cultural characteristics and avoid the ceiling and floor effects in the Chinese population. In 2015, based on the survey data of 3698 Chinese children, the developers carried out a Rasch model analysis of eHCI. The overall Chi-squared fit statistic of fit to the Rasch model was 2078.773 (df 540), *p* < 0.001 and the item fit residual was − 1.1258 (7.6952). The distribution of the item and person locations relative to one another on the same continuum is shown in Fig. 1 (Online). The Person Separation Index (the Rasch equivalent of Cronbach’s alpha indicating level of reliability) was 0.88509, which indicates high reliability. The power of the tests of fit was rated Excellent. The final scale has 62 entries, which can be completed by any person familiar with the child, such as parents, teachers, social workers, etc. The scale includes 9 dimensions: 1) verbal communication, 2) approaches to learning, 3) numeracy and concepts, 4) Reading, 5) writing, 6) cultural identity and spirituality, 7) social-emotional wellbeing, 8) perseverance, and 9) physical health. From these 9 domains an overall literacy and numeracy score is derived, as well as an overall development score, both ranging from 0 to 1 with 1 being the best score.

### Study sample and data collection

This study used data to assess the reliability and validity of the eHCI mainly from the 2016 Shanghai Children’s Health, Education and Lifestyle Evaluation, Preschool (SCHEDULE-P) study. The 2016 SCHEDULE-P study was a cross-sectional survey that investigated the lifestyle, home environment and development of preschool children in 2016. The design, sampling and procedures of the survey have been described previously [20]. A representative sample of newly enrolled preschoolers in Shanghai kindergartens was obtained by stratified random sampling design. There were 20,899 children (age 36–58 months) from 191 kindergartens who enrolled in the study. From these, there were 20,324 families who consented to participate and the parents then completed the online questionnaire of the eHCI. The response rate was 97.2%.

To evaluate the discriminant validity of the eHCI, the demographic information of children and their family was also obtained in the survey. Age and sex of all participants were obtained from the Shanghai Kindergarten Registry Database of the Shanghai Education Committee and further confirmed by parents at the beginning of the study. Maternal education, paternal education, and annual household income were self-reported. The questionnaire included a family assets scale which sought information relating to the number of household cellphones, television, computers, cars and bathrooms [21]. Parents also reported the present height of the child, which was used to evaluate the nutrition status of children. The stunted children were defined as those with height-for-age less than 2 standard deviation using WHO standard for children aged 2–5 years. The principal-factor analysis was conducted to obtain a factor predicting the family assets. This same family asset scale was used in the Program for International Student Assessment (PISA) conducted by Organization for Economic Co-operation and Development (OECD) to reflect family wealth.

To assess how constant eHCI scores remain from one occasion to another, a test-retest reliability survey was conducted in two Shanghai kindergartens not involved in the SCHEDULE-P study. Parents of 183 kindergarten children aged 3–6 years old completed the eHCI for a second time 9.1 [SD: 0.6] days after their first completion. In order to investigate the rater agreement, the eHCI ratings of 168 children from the two kindergartens were also compared between teachers and parent.

To test the correlations between the eHCI and other metrics of ECD by site, we used data from the SCHEDULE-P study, the One Sky program in Ye County and the kindergarten survey in Daming County. The Strengths and Difficulties Questionnaire (SDQ) was used in the SCHEDULE-P study. The Strengths and Difficulties Questionnaire (SDQ) and the Age & Stages Questionnaire: Social Emotional (ASQ: SE), two internationally recognized tools, were reported by parent to assess the psychosocial wellbeing status and the social-emotional development of the child in the SCHEDULE-P study [22]. The One Sky program conducted the study to describe the situation of the left-behind children in Ye County in August 2015. A total of 60 villages were selected from a list of all villages in Ye county provided by the Education Bureau of Ye county and the Bureau of Civil Affairs. All children aged 3 to 4-years and their families were interviewed in these villages. The Age & Stages Questionnaire (ASQ) and the eHCI was filled in by the caregiver of 1918 children. The ASQ was designed to measure child development in the domains of communication, gross and fine motor, problem-solving skills and personal-social skills [23]. The kindergarten survey in Daming County aimed to evaluate the early childhood development of children and the quality of preschool education in rural areas. Sixty-two kindergartens were randomly selected from all 217 kindergartens in Daming County in October 2017. The eHCI was reported by caregivers of all children (age 3–4 years) in the first year of kindergarten. The total sample of 6974 eHCI included 2203 paper and 4744 online questionnaires. The East Asia-Pacific Early Child Development Scales (EAP-ECDS) containing 99 items include seven domains: cognitive development; cultural knowledge and participation; language and emergent literacy, motor development; health, hygiene and safety; socio-emotional development; and approaches to learning [24]. The EAP-ECDS was tested in 1199 children on site by well-trained assessors. When interpreting some of the results presented in this paper it is important to note that for some aspects of the SDQ and the ASQ:SE higher scores represent children with greater development problems, which is opposite to how the other measures of child development are coded. The study was approved by the institutional review Board of the Shanghai Children’s medical center (SCMC), Shanghai Jiao Tong University (SCMCIRB-K2016022–01).

### Statistical analysis

We conducted descriptive analysis on demographic characteristics using the SCHEDULE-P data. Reliability was assessed from analyses of internal consistency, test-retest reliability, and inter-rater agreement. Internal consistency of eHCI was assessed using Cronbach’s coefficient alpha. The intraclass correlation coefficients (ICC) between eHCI scores by parents in two time points were calculated to assess the test-retest reliability. The ICC and paired t-test were calculated separately to assess the agreement and difference of eHCI scores rated by parents and teacher.

To validate the eHCI, we conducted the tests of structure validity, criterion-related validity and discriminant validity. Criterion-related validity of the eHCI was conducted by calculating its correlations with other metrics of ECD. Discriminant validity was tested through multi-level linear regression models assessing score differentials with respect to child sex, parental highest education, family income, quantiles of family assets, and nutrition status. The above analysis was conducted using Stata 14.2 (from StataCorp LP, College Station, Texas, U.S.A.). The Confirmatory Factor Analysis (CFA) model was established to confirm the dimensionality of eHCI. The robust weighted least squares estimator (WLSMV) was used, as the item variables of the eHCI are categorical [25]. The CFA was operated in Mplus 8 (from Muthen & Muthen, Los Angeles, CA, U.S.A.).

## Results

The mean age was 44.3 [SD: 3.6] months. Of those, 52.2% were boys and 47.8% were girls. Sample sizes and weighted demographic characteristics for Shanghai population were presented in Table 1.

**Table 1:** Descriptive characteristics of the sample (Total *n* = 20,324)

Categorical variable	N	%
Sex		
Boys	10,573	52.2
Girls	9751	47.8
Parents’ highest education		
Secondary education and lower (Under Grade 12)	3089	19.7
Tertiary education	13,313	63.3
Post-graduate	3871	17.0
Annual household income		
< 100,000 RMB	4289	26.8
100,000–300,000 RMB	9916	52.1
> 300,000 RMB	4825	21.1
Nutrition status		
Non-stunting	19,851	97.4
Stunting	428	2.6
Continuous variable		Mean ± SD
Age (month)		44.3 ± 3.6
Family assets		−0.1 ± 0.9

N = sample size; Mean / % = weighted mean or percentage adjusting for sampling design

### Reliability

#### Internal consistency

The Cronbach’s α coefficient for Overall Development and Literacy Numeracy were respectively 0.87 and 0.84, and the others for the subscales were presented in Table 2.

**Table 2:** The Cronbach’s α coefficient for eHCI (*n* = 20,284)

	items	Cronbach’s α coefficient
Literacy Numeracy	23	0.84
Overall Development	60	0.87
Subscales		
Verbal	6	0.61
Physical	4	0.18
Approaches	6	0.54
Numeracy Concepts	12	0.75
Reading	8	0.78
Writing	3	0.53
Cultural Spiritual	7	0.71
Social Emotional	10	0.66
Perseverance	4	0.54

*EHCI* Early Human Capability Index

#### Test-retest reliability

As reported in Table 3, the ICC between two scores of Literacy Numeracy and Overall Development in two time points were respectively 0.97 and 0.85, which were interpreted as excellent agreement in temporal stability.

**Table 3:** Intraclass Correlation Coefficients of test-retest and inter-rater for eHCI scores

	Parent test-retest (*n* = 183)	Parent X Teacher (*n* = 168)
Literacy Numeracy	0.97	0.81
Overall Development	0.85	0.63
Subscales		
Verbal	0.71	0.18
Physical	0.77	0.27
Approaches	0.65	0.11
Numeracy Concepts	0.92	0.78
Reading	0.96	0.65
Writing	0.92	0.71
Cultural Spiritual	0.75	0.02
Social Emotional	0.60	0.28
Perseverance	0.48	0.35

*EHCI* Early Human Capability Index

#### Inter-rater reliability

The ICC between parents and teachers were 0.83 and 0.63 for Literacy Numeracy and Overall Development. As reported in Table 3, the lowest agreement between parents and teacher occurred in Approaches and Cultural Spiritual, the highest in the subscales of Numeracy Concepts. The results of the paired t-tests suggested that scores rated by teacher were significantly higher than that by parents for Literacy Numeracy (t = 3.51, df = 167, *P* = 0.001) and Overall Development (t = 2.29, df = 167, *P* = 0.023).

### Validity

#### Factor structure

The confirmatory factor analysis was conducted, and the fit of model was good (χ2 = 509,323, *p* < 0.001; CFI = 0.901; RMSEA = 0.038). Factor loadings of items in each subscale are presented in Table 4. The majority of item’s factor loadings were above 0.7. Only the factor loading of item 50, 51, 55, 56 and 57 was below 0.4, which were all the reverse scored questions.

**Table 4:** Factor loadings of items in each subscale through confirmatory factor analysis (*n* = 20,271)

Items	Verbal	Approaches		Numeracy Concepts	Reading
1	0.622				
2	0.851				
3	0.930				
4	0.842				
5	0.946				
6	0.834				
7		0.411			
8		0.725			
9		0.653			
10		0.474			
11		0.746			
12		0.807			
13				0.751	
14				0.785	
15				0.764	
16				0.701	
17				0.742	
18				0.669	
19				0.658	
20				0.744	
21				0.708	
22				0.605	
23				0.735	
24				0.765	
Items	Writing	Cultural Spiritual	Social Emotional	Perseverance	Physical
25				0.966	
26				0.986	
27				0.847	
28				0.663	
29				0.701	
30				0.597	
31				0.921	
32				0.939	
33	0.726				
34	0.910				
35	0.797				
36		0.665			
37		0.710			
38		0.496			
39		0.795			
40		0.821			
41		0.815			
42		0.800			
43			0.640		
44			0.612		
45			0.768		
46			0.711		
47			0.778		
48			0.731		
49			0.731		
50			0.049		
51			0.160		
52			0.576		
53				0.748	
54				0.890	
55				0.308	
56				0.348	
57					0.020
58					0.636
60					0.650

Notes: Values are factor loadings in confirmatory factor analysis

#### Criterion-related validity

As reported in Table 5, the correlations between the scores of eHCI and other ECD metrics ranged between r = − 0.42 and r = 0.53, and all were statistically significant. The direction of the correlation coefficient and the magnitude of the coefficient were all consistent with expectations, that is the direction of the coding (a high score represents poor development on some scales and low development on others) and the similarity of construct measured by the different instruments.

**Table 5:** Correlations between the scores of eHCI and other ECD metrics

	Measure	Site	Year	N	Raw correlation	
					r	*P* value
Overall development	ASQ	Yexian	2016	1918	0.53	< 0.001
	ASQ:SE	Shanghai	2016	16,478	−0.42	< 0.001
	SDQ	Shanghai	2016	20,277	−0.45	< 0.001
	EAP-ECDS	Daming	2018	1199	0.364	< 0.001
Numeracy and literacy	ASQ	Yexian	2016	1918	0.1254	< 0.001
	ASQ:SE	Shanghai	2016	16,477	−0.24	< 0.001
	SDQ	Shanghai	2016	20,276	−0.26	< 0.001
	EAP-ECDS	Daming	2018	1199	0.445	< 0.001

Notes: *EHCI* Early Human Capability Index, *ECD* Early Child Development, *ASQ* Age & Stages Questionnaire, *ASQ:SE* Age & Stages Questionnaire: Social Emotional, *SDQ* Strengths and Difficulties Questionnaire, *EAP-ECDS* East Asia-Pacific Early Child Development Scales

The SDQ and the ASQ:SE higher scores represent children with greater development problems, which is opposite to how the other measures are coded

#### Discriminant validity

Differences in eHCI scores across several sociodemographic subgroups were shown in Table 6. Girls scored 0.025 (SE: 0.002) higher than boys in Overall Development and 0.017 (SE: 0.002) higher in Literacy Numeracy, adjusted for age, SES factors and nutrition status. Significantly higher scores were achieved by children with higher parental education, and in wealthier families. Compared with those children in normal nutrition status, stunted children scored − 0.04 (SE: 0.006) lower in Overall Development and − 0.03 (SE: 0.009) lower in Literacy Numeracy sifnificantly.

**Table 6:** Associations between demographic and social economic status and eHCI scores

	Overall Development			Literacy Numeracy		
	N	Age-adjusted	Multivariate-adjusted^a^	N	Age-adjusted	Multivariate-adjusted^a^
Sex	20,232			20,231		
boys		Ref.	Ref.		Ref.	Ref.
girls		0.026^***^	0.025^***^		0.019^***^	0.017^***^
		(0.002)	(0.002)		(0.002)	(0.002)
Parental education	20,182			20,181		
Secondary education and lower (Under Grade 12)		Ref.	Ref.		Ref.	Ref.
Tertiary education		0.052^***^	0.036^***^		0.116^***^	0.092^***^
		(0.002)	(0.003)		(0.003)	(0.004)
Post-graduate		0.086^***^	0.059^***^		0.179^***^	0.138^***^
		(0.003)	(0.003)		(0.004)	(0.005)
Annual household income	18,950			18,949		
< 100,000 RMB		Ref.	Ref.		Ref.	Ref.
100,000–300,000 RMB		0.034^***^	0.017^***^		0.071^***^	0.034^***^
		(0.002)	(0.002)		(0.003)	(0.003)
> 300,000		0.067^***^	0.040^***^		0.123^***^	0.068^***^
		(0.002)	(0.003)		(0.003)	(0.004)
Family assets quantile	19,030			19,029		
1[poorest]		Ref.	Ref.		Ref.	Ref.
2		0.016^***^	0.003		0.032^***^	0.004
		(0.002)	(0.002)		(0.003)	(0.003)
3		0.027^***^	0.008^***^		0.049^***^	0.013^***^
		(0.002)	(0.002)		(0.003)	(0.003)
4[richest]		0.036^***^	0.012^***^		0.054^***^	0.010^**^
		(0.002)	(0.002)		(0.003)	(0.004)
Nutrition status	20,227					
Non-stunting		Ref.	Ref.		Ref.	Ref.
Stunting		−0.06^***^	−0.04^***^		−0.08^***^	−0.03^**^
		(0.006)	(0.006)		(0.009)	(0.009)

Values are linear regression coefficients (95% CI)

^a^Adjusted for age and all characteristics included in table

^*^P < 0.05, ^**^P < 0.01, ^***^P < 0.001

## Discussion

The psychometric properties of eHCI were evaluated in a representative sample of children aged 3–4 years from all districts of Shanghai. Results of the present study suggest the eHCI is psychometrically sound for Chinese children.

In terms of reliability indicators, the α coefficient indicates good internal consistency sufficient for group comparison other than the domain of physical [26]. The physical subscale was designed to understand children’s disability, health status and behavior. The four items in the subscale are: “Is this child frequently sickly? “, “Does this child have good hygiene i.e. always wash their hands after toileting?”, “Does this child have any disabilities/special needs?”,“Does this child have a regular diet?”, are not strongly correlated with each other. Perhaps indicating that these physical factors act mainly as independent characteristics rather than as a scale of physical development. An ICC above 0.75 is considered as excellent [27]. The result of our reliability analysis suggested that eHCI had good internal consistency and temporal stability. However, the inter-rater agreement in the present analysis was more variable, with subscales related to Literacy Numeracy showing excellent consistency between scores rated by parents and teachers, and the others showing greater heterogeneity in responses. Since the items in Numeracy Concepts, Reading, and Writing are relatively objective indicators, it is reasonable that scores in those aspects were more consistent between parents and teachers. These results are consistent with the reported reliability of other measures of child development and the reasons for inconsistent are likely to be related to parent, teacher and child factors as well as context (for example; parental knowledge of child development, parent literacy levels, parental engagement in the school system, teacher qualifications and knowledge of development, teachers experience across different socioeconomic settings, child behavior being different in the school compared to home due to shyness or other factors). The paired t-test results suggested that teacher scored higher than parents for the same children. For example, the items in cultural spiritual are “Does this child talk politely?”, and “Is this child good to his or her parents?”. It may be because children act differently in kindergarten than at home. It also may be because parents expected too much of their children. We cannot draw a conclusion without deeper exploration of the reason behind the disagreement. In the future, when using the eHCI or other measures of child development it will be important to distinguish the raters prior to scores being compared across different populations.

The results of confirmatory factor analysis supported the underlying structure of the eHCI. The model fit demonstrated that the extracted factors from all items are capable of assessing the different developmental domains in Chinese children. All but five items have high factor loadings. Those five are reverse coded question: kick, bite or hit adults or other children; impatient; need constant reminding to finish something off; get easily distracted from a task; frequently sickly. Even though the factor loadings of the reverse coded items were lower than expected, it may be important to keep the items worded in a negative fashion. There is evidence to suggest that respondents get into a pattern of response and reversing the direction of a question requires deeper thinking, however others in survey methodology would recommend keeping all survey items in the same direction for simplicity and to reduce confusion [17, 28]. This may be something worth exploring further with future use of the eHCI.

The eHCI showed significant correlations with other metrics covering different domains of child development, such as Age & Stages Questionnaire (gross motor, fine motor, communication, problem-solving, social-personal), Strengths and Difficulties Questionnaire (psychosocial well-being), etc. However, those metrics are inclined to screen the individual with high-risk of development. The eHCI was designed to monitor the comprehensive abilities of children at population level. As such, we would not have expected correlations larger than what was found.

The discriminant validity of eHCI with demographic characteristics was also presented in the results. The eHCI scores of girls were significantly more than those of boys, consistent with the conclusion of other studies that girls mature earlier than boys [29]. The results also suggest that higher eHCI scores appeared in the groups with higher socioeconomic status, in keeping with prior research [30]. A large body of researches has found stunting are negatively related to early childhood development [31, 32], which is also certified using eHCI scale in this study. The significant association between eHCI scores and demographic characteristics verified that eHCI could detect the development heterogeneity of different populations.

This study has several limitations that deserve mention. First, although the eHCI was proved to be a feasible and comprehensive tool for identifying the developmental level of Chinese children, the overall sample was not representative of the national population, even though children from migrant workers from rural areas in Shanghai were included within this sample. Second, although the eHCI could be applied as an instrument for monitoring and to compare the status of early childhood development in different populations worldwide for its cross-culture design, it is not meant to replace traditional screening or diagnostic tools for delayed development. The eHCI emphasizes improving early childhood development at a population level, rather than diagnosing individual children as abnormal. Future studies should take this into consideration according to their target population and goal. Third, although the reliability and validity of eHCI has been tested in this study, there is still no evidence to verify eHCI as a reliable predictor of long-term indicators of academic or working achievement, such as education level, income, and crimes. Longitudinal studies are warranted to test its predictive validity for later outcomes.

## Conclusions

The results of reliability and validity analysis suggest that the eHCI is a valid measurement to assess the overall development of Chinese children aged 3–6 years. It has enabled us to monitor the developmental trajectories of children, implement evidence-based interventions to improve their school readiness, and will ultimately support the evaluation of those interventions. The valuable aspect is that the eHCI can be applied to children from diverse cultural backgrounds, which makes it possible to map the global distribution of early childhood development for allocating scarce resources to help those in greatest demand. In the future, longitudinal studies will be conducted to identify its ability to predict important outcomes in later life.

## Data Availability

The datasets generated and/or analyzed during the current study are not publicly available due to public policy restriction but are available from the corresponding author on reasonable request.

## Abbreviations

ECD: Early Childhood Development
EDI: Early Development Instrument
EHCI: Early Human Capacity Index
SCHEDULE-P: Shanghai Children’s Health, Education and Lifestyle Evaluation, Preschool
SDQ: Strengths and Difficulties Questionnaire
ASQ: SE: Age & Stages Questionnaire: Social Emotional
ASQ: Age & Stages Questionnaire
EAP-ECDS: East Asia-Pacific Early Child Development Scales
ICC: Intraclass correlation coefficients
